# Emergence of the Asian 1 Genotype of Dengue Virus Serotype 2 in Viet Nam: *In Vivo* Fitness Advantage and Lineage Replacement in South-East Asia

**DOI:** 10.1371/journal.pntd.0000757

**Published:** 2010-07-20

**Authors:** Vu Thi Ty Hang, Edward C. Holmes, Duong Veasna, Nguyen Thien Quy, Tran Tinh Hien, Michael Quail, Carol Churcher, Julian Parkhill, Jane Cardosa, Jeremy Farrar, Bridget Wills, Niall J. Lennon, Bruce W. Birren, Philippe Buchy, Matthew R. Henn, Cameron P. Simmons

**Affiliations:** 1 Oxford University Clinical Research Unit, Hospital for Tropical Diseases, Ho Chi Minh City, Viet Nam; 2 Center for Infectious Disease Dynamics, Department of Biology, The Pennsylvania State University, University Park, Pennsylvania, United States of America; 3 Fogarty International Center, National Institutes of Health, Bethesda, Maryland, United States of America; 4 Institute Pasteur in Cambodia, Phnom Penh, Cambodia; 5 Hospital for Tropical Diseases, Ho Chi Minh City, Viet Nam; 6 Wellcome Trust Sanger Institute, Hinxton, United Kingdom; 7 Institute of Health and Community Medicine of the University of Malaysia, Sarawak, Malaysia; 8 Broad Institute, Cambridge, Massachusetts, United States of America; Southwest Foundation for Biomedical Research, United States of America

## Abstract

A better description of the extent and structure of genetic diversity in dengue virus (DENV) in endemic settings is central to its eventual control. To this end we determined the complete coding region sequence of 187 DENV-2 genomes and 68 E genes from viruses sampled from Vietnamese patients between 1995 and 2009. Strikingly, an episode of genotype replacement was observed, with Asian 1 lineage viruses entirely displacing the previously dominant Asian/American lineage viruses. This genotype replacement event also seems to have occurred within DENV-2 in Thailand and Cambodia, suggestive of a major difference in viral fitness. To determine the cause of this major evolutionary event we compared both the infectivity of the Asian 1 and Asian/American genotypes in mosquitoes and their viraemia levels in humans. Although there was little difference in infectivity in mosquitoes, we observed significantly higher plasma viraemia levels in paediatric patients infected with Asian 1 lineage viruses relative to Asian/American viruses, a phenotype that is predicted to result in a higher probability of human-to-mosquito transmission. These results provide a mechanistic basis to a marked change in the genetic structure of DENV-2 and more broadly underscore that an understanding of DENV evolutionary dynamics can inform the development of vaccines and anti-viral drugs.

## Author Summary

Dengue virus (DENV) is a mosquito transmitted RNA virus. One of the most characteristic patterns of DENV evolution is that viral lineages, including whole genotypes, are born and die-out on a regular basis. However, the precise cause of such lineage turnover is unclear. To address this issue we explored the genome-scale genetic diversity and evolution of dengue serotype 2 virus (DENV-2) in Viet Nam, a country with a high burden of dengue disease. We observed that DENV viruses assigned to the Asian 1 lineage were likely introduced into southern Viet Nam in the late 1990's (perhaps from Thailand) and subsequently displaced the resident Asian/American lineage serotype 2 viruses. A similar scenario seems to have occurred in Thailand and Cambodia, such that there appears to have been a region-wide proliferation of Asian 1 lineage viruses. Investigation of Vietnamese patients experiencing DENV-2 infection revealed that Asian 1 viruses also attain higher virus levels in the blood than viruses of the Asian/American lineage. This difference in virus titre is likely to have a profound impact on viral fitness by increasing the probability of mosquito transmission, and therein providing Asian 1 lineage viruses with a selective advantage.

## Introduction

Dengue viruses (DENV) are vector-borne RNA viruses of the family *Flaviviridae* and are classified as either four distinct viruses or different serotypes (DENV-1 to DENV-4), each of which can infect humans. Infection with DENV can cause a spectrum of outcomes, ranging from asymptomatic infection through to clinically significant disease. Severe disease in children is commonly characterised by increased vascular permeability, thrombocytopenia and a bleeding diathesis that leads to life-threatening dengue shock syndrome. Each year, approximately 40 million clinically apparent dengue cases occur globally and an estimated two-thirds of the world's population live in areas of risk [1]. There are currently no licensed dengue vaccines or specific interventions to treat the disease.

The error prone RNA-dependent RNA polymerase responsible for genomic replication is central to the generation of DENV genetic diversity, upon which natural selection can act. Although viral fitness is clearly multi-faceted, selection during DENV evolution is likely to be driven in part by the underlying immune status and DENV-infection history of the human population in which the viruses are circulating. For example, patterns of DHF incidence within endemic populations such as Thailand exhibits complex wave-like dynamics with the four viral serotypes co-circulating in a single population and with each serotype dominant on a 8–10 year periodic cycle [2]. An additional layer of complexity exists in the dynamics of serotype oscillations in that there are multiple, genetically distinct viral lineages or genotypes within each virus serotype [3]. Intriguingly, these viral lineages experience a process of ongoing birth and death, possibly because of fitness differences in the human or mosquito host that allow some lineages to survive better than others. For example, in Thailand a turnover of DENV-2 strains was observed between 1980 and 1987 [4] and of DENV-3 strains in the 1990s [5]. Similarly, within-serotype lineage turnover has been documented in Sri Lanka (DENV-3) [6] and Myanmar (DENV-1) [7]. On a wider scale, the introduction of a genetically distinct Asian/American DENV-2 strain into the Americas resulted in the replacement, and possibly extinction, of the resident American DENV-2 lineage [8], with the process particularly well described in Puerto Rico [9]. Lineage replacement can also have epidemiological significance. For example, the lineage replacement events in the Americas (DENV-2) and Sri Lanka (DENV-3) were associated with increases in disease incidence and severity [6], [8] and thereby implying that intrinsic differences in virulence exist between viruses of the same serotype, a phenomenon consistent with earlier field observations [10], [11].

Although the occurrence of lineage replacement is one the most intriguing aspects of DENV molecular epidemiology, its mechanistic basis is unclear. In particular, it is uncertain whether such lineage replacement events reflect (i) large-scale epidemiological processes that are independent of viral genotype, such as random population bottlenecks, perhaps caused by large-scale declines in mosquito numbers during the annual dry season, or (ii) because the viral lineages in question differ in fitness such that one is able out-compete another, perhaps because they possess mutations that allow them to evade cross-protective herd immunity. Choosing between these two models is of central importance because it enables predictions to be made as to what viral lineages are likely to proliferate in the near future, and in so doing allows the more accurate design of vaccines and anti-viral drugs.

The aim of this study was to reveal, for the first time, the changing transmission patterns of DENV serotypes (and genotypes) in southern Viet Nam and their relationship to disease incidence. Rather than focusing on a single gene in isolation, we undertook an expansive genomic approach, resulting in the largest sample of DENV genome (complete coding region) sequences generated to date. Our results reveal a major lineage replacement event that has occurred within Viet Nam specifically, and in South-East Asia more generally, and which has resulted in the dominance of one viral genotype.

## Materials and Methods

### Patient population and diagnostic investigations

The dengue patients from whom virus genome sequences were determined were enrolled into an ongoing (since 2001) prospective, descriptive study at the Hospital for Tropical Diseases in Ho Chi Minh City, Viet Nam. Patients (or their parents/guardians) gave written informed consent to participate in the study. The study protocol was approved by the Hospital for Tropical Diseases and the Oxford University Tropical Research Ethical Committee. Serological investigations (IgM and IgG capture ELISAs) were performed using paired plasma samples using methods described previously [12]. Serology was interpreted as suggestive of secondary infection if DENV-reactive IgG was detected in the capture ELISA in the first week of illness. DENV viraemia levels were determined using an internally-controlled, serotype-specific, real-time RT-PCR assay that has been validated and described previously [13]. Diagnostic culture for DENV was performed by inoculation of 50 µl of plasma onto C6/36 mosquito cells in plastic 75×12 mm tubes. Cultures were incubated at 30°C for 7 days, then blind passaged twice in C6/36 cells for 7 days each.

### DENV genome sequencing

DENV genomic consensus sequencing was conducted at the Sanger Institute (Cambridge, UK) and the Broad Institute (Cambridge, MA, USA). Two approaches were employed for genomic sequencing. In the first approach (Sanger Institute) (n = 45 viruses), PCR amplification of the DENV-2 genome (C6/36 cells, ≤3 passages) was performed in 3 overlapping amplimers (2.4 kb, 4.5 kb and 4.5 kb, primer sequences available on request). These amplimers were pooled in approximately equimolar concentrations, then randomly sheared by sonication. Sheared DNA fragments were separated according to size by gel electrophoresis and fragments corresponding to a size range of 0.7 to 1.0 kb were removed and shot-gun cloned. From each shotgun library, between 96 and 192 clones were sequenced by dideoxy sequencing using universal and reverse primers. Regions of low or no coverage we filled by specific PCR amplification and sequenced.

In a second approach, viral genomes (Viet Nam, n = 142 and Cambodia n = 39) were sequenced using the Broad Institute's capillary sequencing (Applied Biosystems) directed amplification viral sequencing pipeline (see http://www.broadinstitute.org/annotation/viral/Dengue). This sequencing effort was part of the Broad Institute's Genome Resources in Dengue Consortium (GRID) project. Viral RNA was isolated from diagnostic plasma samples (QIAmp viral RNA mini kit, Qiagen) and the RNA genome reverse transcribed to cDNA with superscript III reverse transcriptase (Invitrogen), random hexamers (Roche) and a specific oligonucleotide targeting the 3′ end of the target genome sequences (nt 10701–10723 for DENV-2). cDNA was then amplified using a high fidelity DNA polymerase (pfu Ultra II, Stratagene) and a pool of specific primers to produce 14 overlapping amplicons of 1.5 to 2 kb in size for a physical coverage of 2X. Amplicons were then sequenced in the forward and reverse direction using primer panels consisting of 96 specific primer pairs, tailed with M13 forward and reverse primer sequence, that produce 500–700 bp amplicons from the target viral genome. Amplicons were then sequenced in the forward and reverse direction using M13 primer. Total coverage delivered post amplification and sequencing was ∼8-fold. Resulting sequence reads were assembled *de novo* and annotated sing the Broad Institute's in-house viral assembly and annotation algorithms. All genome sequences newly determined here have been deposited in GenBank and assigned accession numbers (Table S1).

### E gene sequencing

In brief, the virus nucleotide sequence from position 817–2520 was amplified in 5 overlapping amplimers of between 403 and 503 nt in length. Amplification was by RT-PCR using a high fidelity polymerase and RNA extracted directly from patient plasma samples as template. Each amplimer was sequenced on both strands by conventional capillary sequencing on an Applied Biosystems 3130 genetic analyzer using specific primers. The resulting sequence reads were assembled and the E gene sequence annotated in AlignX, a software program in the vectorNTI suite (Invitrogen, USA). Primers used for sequence amplification are available from the authors on request. All E gene sequences newly determined here have been deposited in GenBank and assigned accession numbers; GU211738-GU211764, GU434146-GU434159 and GU908494- GU908520).

### Evolutionary analysis

A sequence alignment was manually constructed (in Se-Al, v2.0) for the complete coding regions (10173 nt) of 187 DENV-2 genome sequences upon which phylogenetic analysis could be undertaken. In addition, to place these data in a wider phylogenetic context, we extracted the envelope (E) gene region from these genomic sequences and combined these data with all those E gene sequences sampled globally and available on GenBank. This resulted in a data set of 941 E gene sequences, 1485 nt in length.

For the 941 E gene sequences we utilized the maximum likelihood (ML) approach available within the PHYML package [14] and incorporating the GTR+Γ_4_ model of nucleotide substitution. A bootstrap resampling process (1,000 replications) using the neighbor-joining method available in the PAUP* package [15] was used to assess the robustness of individual nodes on the phylogeny, also employing the GTR+Γ_4_ substitution model and using the parameter settings taken from PHYML. A very similar phylogeny containing the same major groupings was obtained using the ML method within the GARLI package and assuming the simpler HKY85+Γ_4_ model [16] (tree available from the authors on request).

To place the evolution of the Vietnamese DENV-2 in real time we inferred a Maximum Clade Credibility (MCC) tree of the 187 complete genome sequences using the Bayesian Markov Chain Monte Carlo (MCMC) method available in the BEAST package [17] and employing the exact day of viral sampling. This analysis utilized a strict molecular clock, a GTR+Γ_4_ model of nucleotide substitution, a different substitution rate for each codon position, and a Bayesian skyline prior as the latter is clearly the best descriptor of the complex population dynamics of DENV. Similar results, with no major differences in topology or coalescent times, were obtained under a relaxed (uncorrelated lognormal) molecular clock and different substitution models (results available from the authors on request). All chains were run for a sufficient length (usually 300 million generations) and multiple times to ensure convergence with 10% removed as burn-in. This analysis also allowed us to estimate times to common ancestry (TMRCA) for key nodes on the DENV-2 phylogeny. The degree of uncertainty in each parameter estimate is provided by the 95% highest posterior density (HPD) values, while posterior probability values provide an assessment of the degree of support for each node on the tree. The BEAST package was also used to infer Bayesian skyline plots for both the Asian I (n = 139) and Asian/American (n = 46) genotypes. This analysis enabled a graphical depiction of changing levels of relative genetic diversity through time (N_e_τ, where N_e_ is the effective population size and τ the host-to-host generation time).

### Preparation of virus for *Ae. aegypti* infection

Low passage (<4) virus stocks of DENV-2 isolates were prepared on C6/36 cell cultures and quantified by plaque assay on BHK-21 cells with the titre calculated in plaque forming units per ml. All virus inocula for mosquito studies were prepared by infection of C_6_/36 cells (MOI = 0.1) followed by culture for 4 days at 28°C. Cultures were centrifuged and the culture supernatant used to spike artificial blood meals as described below.

### *Ae. aegypti* and DENV infection

*Ae. aegypti* from immatures (larvae/pupae) sampled from 5 discrete locations in Ho Chi Minh City (HCMC), Viet Nam during May 2009 were used for generation of low filial number mosquitoes for oral infection studies. Adults were allowed to emerge in the laboratory, mate randomly, and feed on fresh defibrinated sheep blood meal held at 37°C through pieces of parafilm stretched over water-jacketed glass feeders. Adults were allowed to lay eggs on wet filter papers which were then collected and stored on dry pieces of filter paper maintained under high humidity. Larvae were reared on a diet of commercial dog food. Pupae were transferred to screened cages, and adults were fed 10% sucrose in PBS ad libitum. All mosquitoes were maintained in an insectary at 27°C, with a relative humidity of 80% and a 12∶12 light-dark cycle in an environmental chamber. In order to obtain a large number of F_2_ females, egg batches from multiple gonotrophic cycles were combined and hatched simultaneously.

Artificial blood meals consisted of fresh, defibrinated sheep blood to which serial, ten-fold dilutions of culture supernatant from individual DENV-2 infected C6/36 cells was added at a final dilution of 1/5 per blood meal. Cohorts of 40, 7 day-old female mosquitoes starved of sucrose for 18–24 hrs were offered an infectious blood meal containing either neat or diluted (10^−1^, 10^−2^, 10^−3^) virus culture supernatant for 45 min via membrane feeding as described above. Fully engorged mosquitoes were collected and incubated for 12 days. After 12 days mosquitoes were killed by incubation at −20°C for 1 hr and the mosquito carcass from each individual placed into 0.7 ml of mosquito diluent (MD) consisting of 20% heat-inactivated fetal bovine serum in PBS with 50 µg/ml penicillin/streptomycin, 50 µg/ml gentamicin, and 2.5 µg/ml fungizone. All samples were stored at −80°C before processing. Samples were thawed on ice and homogenized. Infected bodies were detected by quantitative RT-PCR (qRT-PCR) on 140 µl of homogenate (experiment 1) or by NS1 detection using a commercial antigen capture ELISA (BioRad) and 50 µl of homogenate (experiment 1 and 2). NS1 detection was 100% specific and 97% sensitive relative to qRT-PCR for detection of infected carcasses (Simmons et al, unpublished results). The 50% infectious dose values were determined using a three parameter dose-response curve fitted in Prism software (Version 5, Graphpad Software Inc, La Jolla, CA).

### Statistical analysis

All statistical analysis was performed using Intercooled STATA version 9.2 (StataCorp, TX). Significance was assigned at *P*<0.05 for all parameters and were two-sided. Uncertainty was expressed as 95% confidence intervals. The Mann Whitney test was used for continuous variables. In addition, we compared log10-viremia levels between the two-genotypes using a multiple linear regression model. In addition to the genotype, we adjusted for the following potential confounding factors: age, sex, infection status (primary, secondary, or unknown), and day of illness.

## Results

### Emergence of the Asian 1 lineage of DENV-2 in southern Viet Nam

**Figure 1:**
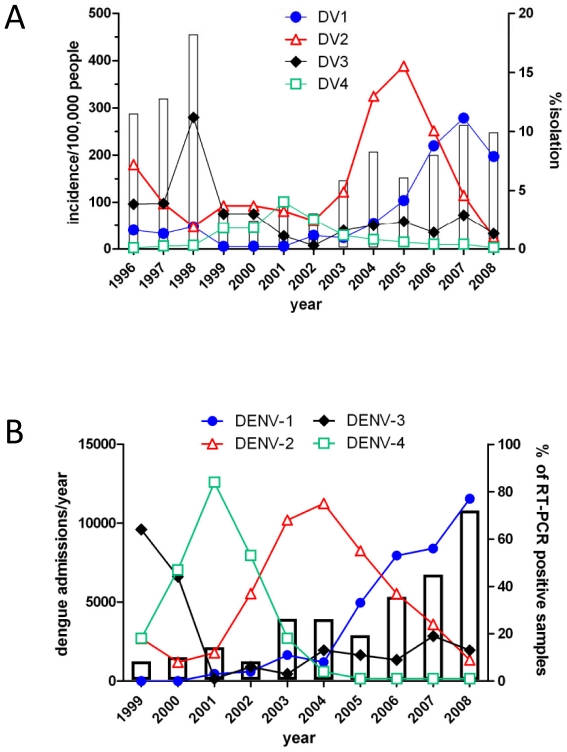
Fluctuations in dengue incidence and serotype abundance in southern Viet Nam. **A**) Changes in the incidence of hospitalized dengue cases (left-hand axis, open bars) and the relative virus prevalence (right hand axis) between 1996 and 2008 in the southern 20 provinces of Viet Nam. Data is from the Vietnamese Ministry of Health Dengue passive surveillance scheme and kindly provided by the Pasteur Institute, HCMC, Viet Nam. **B**. Case burden (left-hand axis, open bars) and DENV serotype prevalence detected by RT-PCR in children and adults (n = 3463 RT-PCR positive patients) enrolled into clinical studies at the Hospital for Tropical Diseases, Ho Chi Minh City between 1999 and 2008. The Hospital for Tropical Diseases is a tertiary referral hospital for infectious diseases. The values underneath the graph represent the number of RT-PCR positive samples each year.

Dengue is hyperendemic in southern Viet Nam. A Ministry of Health coordinated clinical and virological surveillance system operates in the 20 southern provinces of Viet Nam and has revealed oscillations in disease incidence and serotype prevalence in this region since 1996 (Fig. 1A). During 2003–2006, DENV-2 was the most prevalent serotype detected by surveillance and its circulation was temporally associated with increased disease incidence relative to the period 1999–2002 (Fig. 1A). Correspondingly, between 2003–2004, and to a lesser extent 2005–2006, the Hospital for Tropical Diseases in Ho Chi Minh City (HCMC) experienced an increased dengue case burden relative to previous years, most of which was attributable to DENV-2 infection (Fig. 1B).

**Figure 2:**
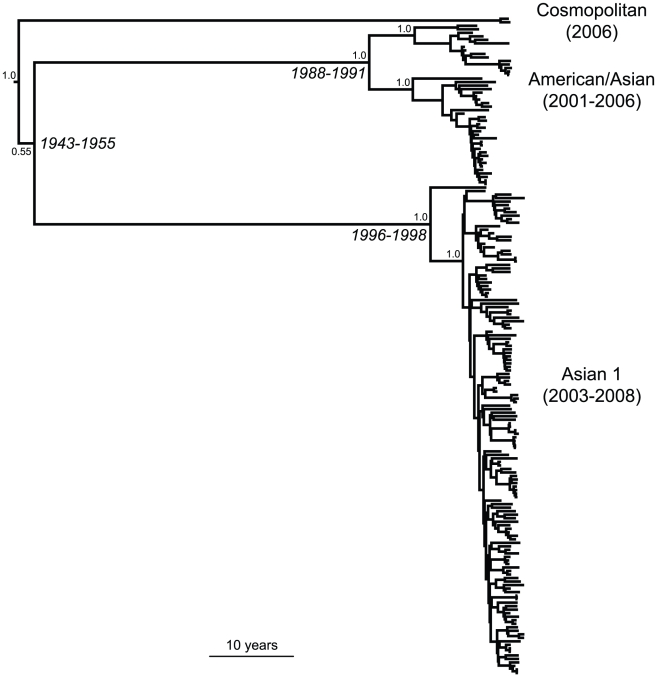
Maximum clade credibility (MCC) tree of complete coding region sequences of DENV-2 sampled in Viet Nam. The major genotypes of DENV-2 and their sampling times (in parentheses) are marked. The 95% credible values for the time to the most recent common ancestor of the Asian I and Asian/American genotypes in Viet Nam are shown. In all cases tip times reflect the day of sampling of each virus. The tree is automatically rooted under the assumption of a molecular clock.

To understand the evolutionary background to the emergence and decline of DENV-2, we determined the complete coding region (consensus) sequence of this virus from 187 hospitalized patients with residential addresses in or around HCMC and sampled between 2001 and 2008 (accession numbers in Table S1). In total, 143 (77%) coding region sequences were obtained directly from patient plasma, 2 (1.0%) from cerebrospinal fluid and 42 (22%) from cultured virus (all with ≤3 passages in C6/36 cells). Phylogenetic analysis revealed that three major lineages of DENV-2 in Viet Nam, representing the Cosmopolitan (n = 2), Asian/American (n = 46) and Asian 1 (n = 139) genotypes, were present in the sampled population (Fig. 2). The complete coding region phylogeny was also marked by a strong temporal structure, such that viruses belonging to the Asian/American genotype were only sampled between 2001 and 2006, and not thereafter (Fig. 2). In contrast, Asian 1 viruses were only sampled from 2003 onwards but quickly became the dominant genotype in the DENV-2 population (Fig. 2). Bayesian molecular clock analysis suggested the most recent common ancestor of Asian 1 viruses in southern Viet Nam existed between 1996–1998. In contrast, the most recent common ancestor of Asian/American lineage viruses may have originated more than a decade earlier, with a credible range of dates spanning 1988–1991. The dramatic emergence and then dominance of Asian 1 viruses is also apparent on Bayesian skyline plots of viruses from each genotype (Fig. S1). The skyline plot for the Asian/American genotype is characterized by a down turn in relative genetic diversity in recent years, indicative of a decline of incidence, while the equivalent skyline plot for Asian I genotype exhibits both continued growth and a higher level of relative genetic diversity, itself compatible with elevated fitness (Fig. S1). Genome-wide rates of evolutionary change were similar in both viruses at ∼1×10^−3^ nucleotide substitutions per site, per year. Importantly, the temporal structure of the DENV-2 tree was not related to spatial differences in sampling, with viruses belonging to both clades being sampled from the same geographical area (Fig. S2).

### E gene phylogeny and temporal patterns of genotype dominance in Viet Nam, Cambodia and Thailand

**Figure 3:**
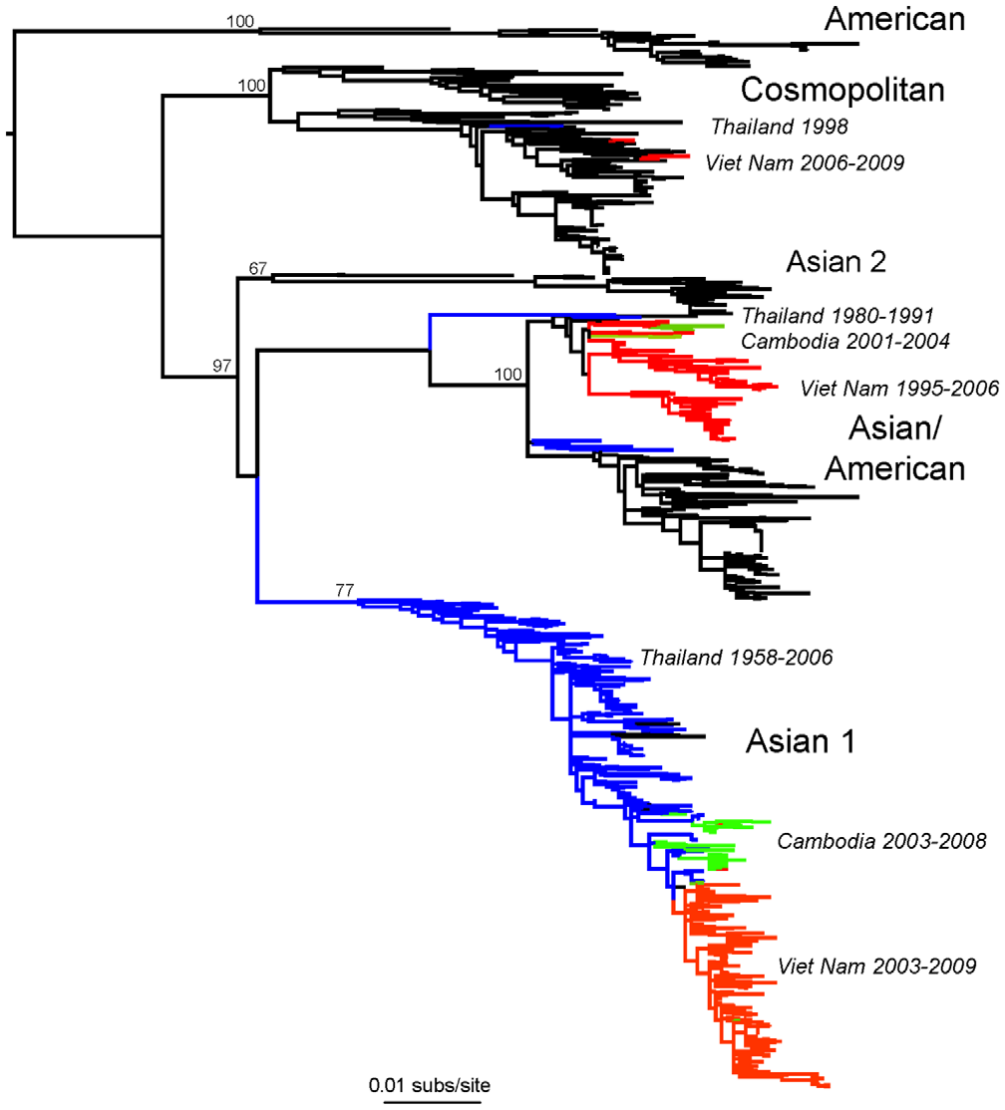
Maximum likelihood tree of 941 DENV-2 E gene sequences sampled globally. Viruses from Viet Nam are shaded red, those from Cambodia shaded green and those from Thailand shaded blue. The sampling years for Viet Nam, Cambodia and Thailand are annotated next to the tree. Bootstrap support values are shown at key nodes. The tree is mid-point rooted for purposes of clarity and all horizontal branch lengths are drawn to a scale of nucleotide substitutions per site.

**Figure 4:**
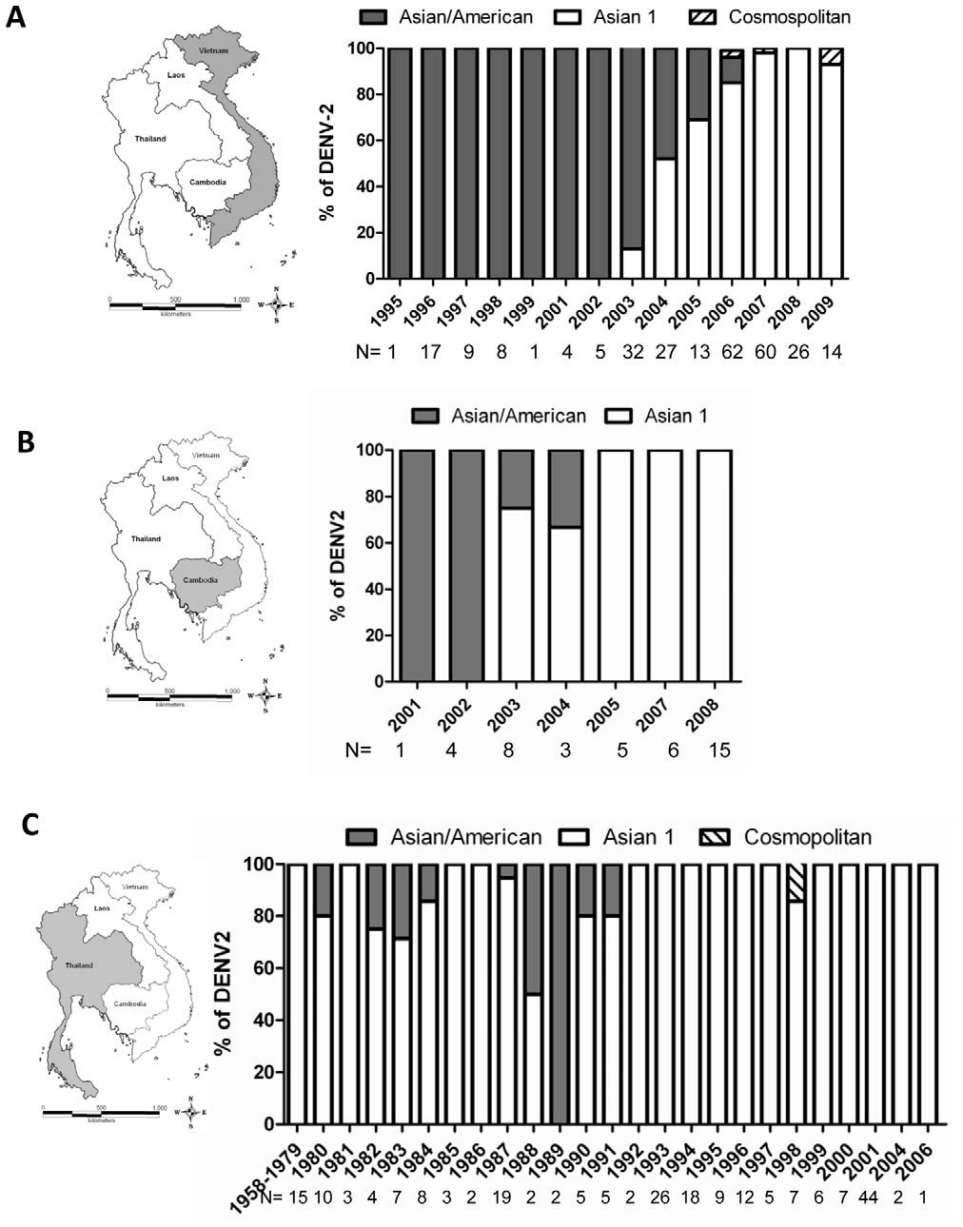
Temporal pattern of DENV-2 genotype replacement in Viet Nam, Cambodia and Thailand. The relative proportion of sampled DENV-2 viruses belonging to either Asian I or Asian/American genotypes (as determined in Fig. 3) is shown by year of sampling in **A**) Viet Nam, **B**) Cambodia and **C**) Thailand. The values below the graph represent the number of E gene sequences sampled per year.

To take a more expansive view of the DENV-2 virus population, we performed a phylogenetic analysis of all globally sampled E gene sequences currently available. This included 68 new E gene sequences generated in the course of this study from DENV-2 viruses sampled from patients in HCMC, Viet Nam between 1995–2009. Furthermore, we included 39 sequences from DENV-2 isolates sampled as part of the Cambodian national dengue surveillance program and also generated in this study (Table S1). Phylogenetic analysis indicated that all available Vietnamese DENV-2 E gene sequences sampled between 1995 to 2002 (n = 45) belonged to the Asian/American genotype (Fig. 3), with Asian 1 viruses being first sampled (by this study) in 2003 and dominating after 2006. The temporal structure in the both the genome (Fig. 2) and E gene trees (Fig. 3), together with the Bayesian molecular clock analysis, supports the contention that Asian 1 genotype viruses were a relatively recent introduction into southern Viet Nam, or circulated at a level below the sensitivity of sampling prior to 2003. Strikingly, there are distinct similarities in the temporal structure of the Vietnamese and Cambodian E gene phylogenies, with Asian 1 genotype viruses also being first sampled in Cambodia in 2003 and apparently displacing the resident Asian/American genotype to the extent that only Asian 1 viruses were sampled in Cambodia after 2005 (Fig. 3). A similar pattern is evident in Thailand, for which sampling is most intense (n = 225), and where both Asian 1 and Asian/American genotype viruses apparently co-circulated in the 1980's, only for Asian 1 viruses to then entirely dominate the sampling since 1991 (Fig. 3). Overall, it is striking that Asian 1 genotype viruses now dominate the sampled DENV-2 population in these three high burden countries. The temporal pattern by which Asian/American and Asian 1 viruses were sampled in Viet Nam, Cambodia and Thailand is summarized in Figure 4. Given the timing and high frequency with which Asian I viruses are sampled in Thailand it is possible that this represents the source population for this genotype, although this will need to be confirmed on a more geographically balanced sample of sequences. More broadly, this E gene phylogenetic analysis reveals that no DENV-2 from South-East Asia sampled after 2006 belonged to the Asian/American genotype, clearly indicating that this viral lineage has suffered a major decline in population frequency in this region. Finally, it is striking that although only four Vietnamese viruses fell into the Cosmopolitan genotype on the basis of E gene sequence data, three of these, sampled from HCMC during the period 2006–2009, clustered together on the tree (Fig. 3). Hence, despite the dominance of Asian I genotype, “refugia” of other viral lineages can persist in localities with large numbers of susceptible hosts.

### Genetic features of Vietnamese Asian 1 genotype viruses

The branch separating the Vietnamese Asian/American and Asian 1 viruses was characterized by 668 nucleotide and 73 amino acid differences, of which 16 amino acid differences can be considered non-conservative (Table 1). Of the 73 amino acid differences, only three (NS3_142K-R_, NS3_186R-K_ and NS5_563K-R_) occurred in previously mapped T cell epitopes [18], suggesting these viruses could have very similar antigenic characteristics for T cells. Two non-conservative amino acid replacements in the E protein – at E226 (T to K) and E228 (G to E) – are of particular note as they had not been previously detected in any of >2000 DENV E gene sequences available on GenBank and occur at amino acid sites that are largely invariant at the global scale. Such an evolutionary pattern is strongly suggestive of a major impact on fitness and it is notable that these changes fall in a surface exposed loop in the E protein [19]. Experimentally determining the fitness effects of these mutations, and perhaps of other that define the Asian I genotype, is clearly an important avenue of future research.

**Table 1:** Amino acid changes that distinguish the Vietnamese Asian/American and Asian 1 genotype polyprotein sequences and that represent a difference in charge or side chain.

Gene	Amino acid residue	Asian/American genotype	Asian I genotype
prM/M	16	R	I^b^
E	83	N	K^a^
E	203	D	N^a^
E	226	T	K^a^
E	228	G	E^a^
E	346	H	Y^a^
NS1	50	H	Q^a^
NS1	80	S	A^b^
NS1	264	T	I^b^
NS2A	153	S	L^b^
NS2A	189	A	T^b^
NS4B	22	E	Q^a^
NS5	135	T	I^b^
NS5	175	D	N^a^
NS5	196	T	A^b^
NS5	865	T	A^b^

a Altered amino acid charge.

b Altered amino acid side chain.

### Asian 1 versus Asian/American lineage viruses: growth in mosquito cells and infectivity in local *Ae. aegypti* mosquitoes

To explore fitness differences in the mosquito host, we first compared the growth dynamics of three viruses from each of the Asian 1 and Asian/American genotypes in *Ae. albopictus* C6/36 cells *in vitro*. At multiplicities of infection of 0.1 and 0.01 we did not detect measurable differences in the rate of virus replication or the peak viral titre attained after 6 days of culture (Fig. S3). The 50% infectious dose (ID_50_) of 3 low-passage virus isolates from each DENV-2 lineage was then measured in low filial cohorts of *Ae. aegypti* to determine if Asian 1 viruses had a measurable advantage in “infectiousness”. The *Ae. aegypti* mosquitoes used in these experiments were F2 generation derived from immature forms collected in HCMC, Viet Nam. Twelve days after ingesting a spiked-blood meal, DENV infection was determined by qRT-PCR and detection of NS1 in homogenates of individual mosquito carcasses. The ID_50_ values determined for each virus were consistent between independent experiments, but there was substantial variation between viruses from the same lineage and there was no apparent trend towards lower ID_50_ values for Asian 1 lineage viruses (Table 2). These data suggest that infectiousness *per se* for local *Ae. aegypti* might not account for the epidemiologically observed fitness differences between these viral lineages.

**Table 2:** The 50% infectious dose (ID_50_)(pfu/ml) of three different virus isolates from the Asian 1 and Asian/American genotypes for female *Ae. aegypti* mosquitoes.

Strain name	GenBank accession number	genotype	Log10 ID_50_ a pfu/ml experiment 1	Log10 ID_50_ a pfu/ml experiment 2	Total no of mosquitoes assessed
DENCO-33/55	EU482542	Asian I	4.09	4.86	81
DENCO-31/212	EU482659	Asian I	5.13	5.22	75
DENCO-31/178	EU482654	Asian I	>7	ND b	106
DF-670	FM210219	Asian/American	>5.3	>7	77
DF-699	FM210221	Asian/American	5.36	5.78	114
DF-726	FM210209	Asian/American	4.55	4.84	108

a Female mosquitoes (7-10days old) were provided a blood meal spiked with 10-fold serial dilutions (4 dilutions per virus) of the indicated virus isolates. Mosquito carcasses were assessed for infection 12 days post-feeding. *Ae. aegypti* fed blood meals to which sterile culture media, or heat-inactivated DENV-2, had been added were uninfected 12 days later.

b Not done.

### Clinical and virological features of Asian/American or Asian 1 DENV-2 infection in Vietnamese children

**Figure 5:**
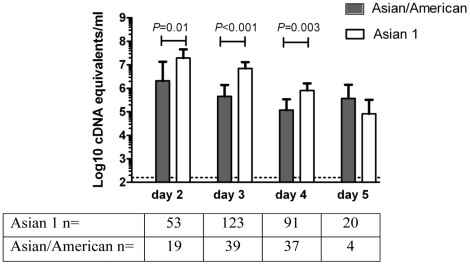
Viraemia levels in Asian 1 and Asian/American genotype infections by day of illness. Shown are the mean (±95% confidence intervals) viraemia levels measured at the time of study enrolment in pediatric dengue cases infected with either Asian 1 (n = 289) or Asian/American (n = 100) genotype viruses. All patients were enrolled in the same study ward and values below the graph represent the number of cases enrolled by duration of illness. Viraemia levels were significantly higher in Asian 1 infections in patients enrolled on day 2, 3 or 4 of illness (t test). Data for patients sampled on day 1 of illness is not shown because of the small sample size (N = 3).

We hypothesized that the rapid displacement of Asian/American viruses by Asian 1 viruses in Viet Nam reflected a relative fitness advantage that might manifest as a measurable virological feature in patients with dengue. To explore this further, the genotype of DENV-2 virus in 389 pediatric inpatients with DENV-2 infections and admitted to the Hospital for Tropical Diseases between 2001 and 2008 was determined by sequencing of nucleotides 9938-10115 (accession numbers GU211349-GU211737) and alignment with reference sequences. All patients were in a prospective study of dengue and admitted to the same pediatric ward. The serological and demographic and features were not significantly different between the two groups (Table 3). Fever clearance times (a surrogate of duration of viral infection) did not differ significantly between patients with Asian 1 (n = 289) or Asian/American (n = 100) DENV-2 infections (*P* = 0.27, log-rank test), nor did platelet nadirs (*P* = 0.07, Mann Whitney test) or maximum haematocrit levels (*P* = 0.16, Mann Whitney test). Intriguingly, however, viremia levels were significantly higher in patients with Asian 1 DENV-2 infections compared to Asian/American DENV-2 infections at the time of study enrolment (Fig. 5). In a multivariate analysis, log10-viremia levels were significantly higher for the Asian 1 genotype at the time of study enrolment: the adjusted mean difference between the two genotypes was 0.94 (95% CI 0.60 to 1.27; p<0.001). Higher viremias in Asian 1 DENV-2 infections could plausibly lead to an increased rate of human-to-mosquito transmission and hence an elevated fitness.

**Table 3:** Baseline characteristics of children with Asian 1 or Asian/American DENV-2 infections at the Hospital for Tropical Diseases 2001–2008.

	Asian I (n = 289)	Asian/American (n = 100)
median age (range)	12 (5–15)	12 (5–16)
Sex (no. male, %)	172 (59.2%)	46 (46%)
median day of illness (range)	3 (1–5)	3 (1–5)
Year of enrolment (no., %)	2001: 0 (0%)2002:0 (0%)2003∶40 (13.8%)2004∶38 (13.1%)2005∶32 (11.1%)2006∶86 (29.8%)2007∶81 (28%)2008∶12 (4.2%)	2001∶2 (2%)2002∶10 (10%)2003∶52 (52%)2004∶15 (15%)2005∶14 (14%)2006∶6 (6%)2007∶1 (1%)2008:0 (0%)
primary infection (no., %)	20 (6.9%)Missing data n = 7	8 (8%)Missing data n = 6
Fever clearance time^a^ (median, interquartile range)	5 (5–6)	5 (5–6)
Maximum hematocrit^b^ (median, interquartile range)	45 (42–49.1)	44.5 (41.5–48)
Platelet nadir^c^ (median, IQR)	56000 (34500-90000)	49000 (28000–8000)
Resident in HCMC	219 (75.8%)	82 (82%)

a Time to defervescence from the self-reported day of fever onset.

b Maximum hematocrit recorded during hospitalization.

c Lowest platelet count recorded during hospitalization.

## Discussion

Oscillations in dengue incidence and serotype prevalence are a characteristic feature of the epidemiology of this virus. Although changes in genotype composition through time are similarly a relatively common observation in studies of DENV evolution, to date there has been generally insufficient data to determine (i) whether changing patterns of genotype prevalence have any association with viral phenotype including the virological manifestation of DENV infection, and (ii) the evolutionary basis of these lineage replacement events, and specifically the respective roles of natural selection versus genetic drift.

Here we demonstrate a rapid and apparently complete lineage (genotype) replacement event within DENV-2 in southern Viet Nam that was temporally associated with an increase in disease incidence. Strikingly, a functional basis for the displacement of resident Asian/American lineage viruses was suggested by higher viremia levels in pediatric patients with Asian 1 DENV-2 infections. The presence of higher viremias in children hospitalized with Asian 1 DENV-2 infections relative to Asian/American DENV-2 infections would likely increase the probability of human-to-mosquito transmission and hence facilitate greater population diffusion. Another possible outcome of higher viraemia levels is an increased incidence of more severe disease. We did not detect significant differences in the extent of capillary permeability or thrombocytopaenia between patients with Asian 1 or Asian/American viruses in the cohort of hospitalized patients (n = 389) here. This suggests that Asian 1 DENV-2 infections were not overtly associated with more severe disease. However, this was a relatively small sample size to detect differences in clinical outcomes and a larger cohort of symptomatic patients, including non-hospitalised individuals, would most likely be needed to answer this question definitively.

Our best estimates suggest the Asian 1 genotype was first introduced into southern Viet Nam in the late 1990's. Our sampling illuminated the replacement of the previously dominant Asian/American genotype by Asian 1 viruses during 2003–2007, a period in which there was an almost doubling of dengue incidence, mostly associated with DENV-2.

A large number of susceptible hosts in the population, and an associated increased force of infection, could help explain the seemingly short period in which genotype replacement occurred. Whether the apparent fitness advantage of Asian 1 viruses could be attributable to “antigenic fitness” in the face of the population-wide immune landscape during this period is unknown. However, there is a precedent for biologically relevant antigenic differences between genotypes of DENV-2. For example, South-East Asian DENV-2 viruses are less susceptible than American lineage viruses to cross-neutralization by antibodies elicited by DENV-1 infection [20]. Population wide seroepidemiology, coupled with a better understanding of correlates of immunity, are clearly needed to understand serotype and genotype replacement in all endemic regions.

Virus traits in the mosquito host might also explain the difference in fitness between the Asian I and Asian/American genotypes. As an example, previous studies have demonstrated “SE Asian genotype” viruses (there was no analysis of differences between Asian 1 or Asian/American genotypes) are more infectious and disseminate faster in *Ae. aegypti* mosquitoes, and replicate more efficiently in human dendritic cells, than American genotype DENV-2 viruses [21], [22], [23]. However, we were unable to detect a measurable difference between Asian 1 or Asian/American viruses in terms of overall replication rates in C6/36 mosquito cells, or infectiousness for local *Ae. aegypti* mosquitoes. Other features of the virus-mosquito interaction (e.g. extrinsic incubation time) could be equally or more important than the infectious dose. However, published data from Armstrong *et al.* suggested that Asian 1 and Asian/American viruses had similar dissemination rates in *Ae. aegypti* mosquitoes [22]. Further studies are therefore needed to understand the importance of the mosquito as a site for the expression of the fitness differences between these two virus lineages.

Intriguingly, the displacement of Asian/American lineage viruses by Asian 1 viruses has also seemingly occurred in Thailand and Cambodia. In Thailand, the Asian/American genotype most likely co-circulated with the Asian 1 genotype for at least a decade prior to 1991, but is then absent from amongst the 139 Thai DENV-2 viruses sampled between 1992 and 2006, which all belong to the Asian 1 lineage. In Cambodia, despite a smaller sample size, lineage replacement appears to have occurred along a remarkably similar time-frame to that seen in Viet Nam, with only Asian 1 viruses being sampled after 2005. Both Thailand and Cambodia have considerable transport links with Viet Nam and it's conceivable these are sources for the introduction of Asian 1 viruses into Viet Nam.

In sum, we show that a lineage replacement event in DENV is highly likely to be linked to an underlying difference in fitness. This suggests that natural selection may play a more important role in shaping viral dynamics than previously realized. The virus genetic traits associated with the fitness of Asian 1 viruses are difficult to identify definitively on the basis of sequence data alone. Elucidation of the possible functional consequences of the 16 amino acid differences that characterize the Asian I viruses will clearly require complex reverse genetic experiments. However, we predict that the amino acid changes at E226 and E228 will be of particular importance given that they occur at sites that are invariant across all DENV sequences sampled to date (and which suggest that the vast majority of mutations at these sites are strongly deleterious because they have a major impact on fitness). Our documentation of a major lineage replacement event, coupled with the current dominance of Asian I viruses, suggests that this genotype will continue to dominate DENV-2 infections in Thailand, Cambodia and Viet Nam unless there is a major change in the host environment, such as that brought on by changes in serotype (and which themselves exhibit complex population dynamics [24]).

The prevalence of Asian I DENV-2 viruses has multiple implications. First, it is paramount that the DENV-2 component of future dengue vaccines (reviewed in [25]) be competent at eliciting immunity to viruses belonging to this genotype. Similarly, programs to develop anti-viral drugs for dengue should include Asian 1 DENV-2 viruses in their pre-clinical discovery and development programs [26]. Furthermore, we would predict that Asian 1 viruses will continue to outcompete Asian/American DENV-2 viruses. A likely future setting for this event is in the Americas where currently Asian/American DENV-2 viruses predominate, having themselves displaced the American genotype.

## Supporting Information

Figure S1 Bayesian skyline plots, showing changes in relatively genetic diversity through time (N_e_τ), for the Asian/American (n = 46) and Asian I (n = 139) genotypes in Viet Nam. Time (x-axis) is measured in days from the present (day 0), although it is important to note that different time-scales are used for each genotype. Note the decline of the Asian/American genotype correspondent to the rise of Asian I.
(9.96 MB TIF)

Figure S2 Map showing the provinces of southern Viet Nam. The numbers in each province represent the number of patients who reported living in that province at the time of admission to hospital and from whom a DENV-2 genome sequence was determined.
(8.24 MB TIF)

Figure S3 Growth kinetics of Asian/American and Asian 1 DENV-2 viruses in C6/36 cells. Three low-passage viruses representing Asian 1 (GenBank accession numbers: EU482542, EU482659, EU482654) and Asian/American genotype viruses (GenBank accession numbers: FM210221, FM210219, FM210221) were inoculated onto C6/36 cells in 2 ml tubes at multiplicities of infection of 0.1 and 0.01. Ten minutes after inoculation (time point = 0), and again every 24 hrs, the culture supernatant was sampled (100 microlitres) and the virus titre determined by plaque titration on BHK-21 cells. Shown are the mean and 95% confidence interval of the titre by day of collection. The dashed line represents the limit of detection.
(0.11 MB TIF)

Table S1 Genbank Accession numbers of DENV-2 genome sequences determined in this study.
(0.22 MB DOC)

Checklist S1 STROBE checklist.
(0.09 MB DOC)
