# Supplementary figures and images for: Emergence of the Asian 1 Genotype of Dengue Virus Serotype 2 in Viet Nam: In Vivo Fitness Advantage and Lineage Replacement in South-East Asia
Source: PLoS Negl Trop Dis. 2010 Jul 20;4(7):e757. doi: 10.1371/journal.pntd.0000757 (PMC2907417; doi:10.1371/journal.pntd.0000757)

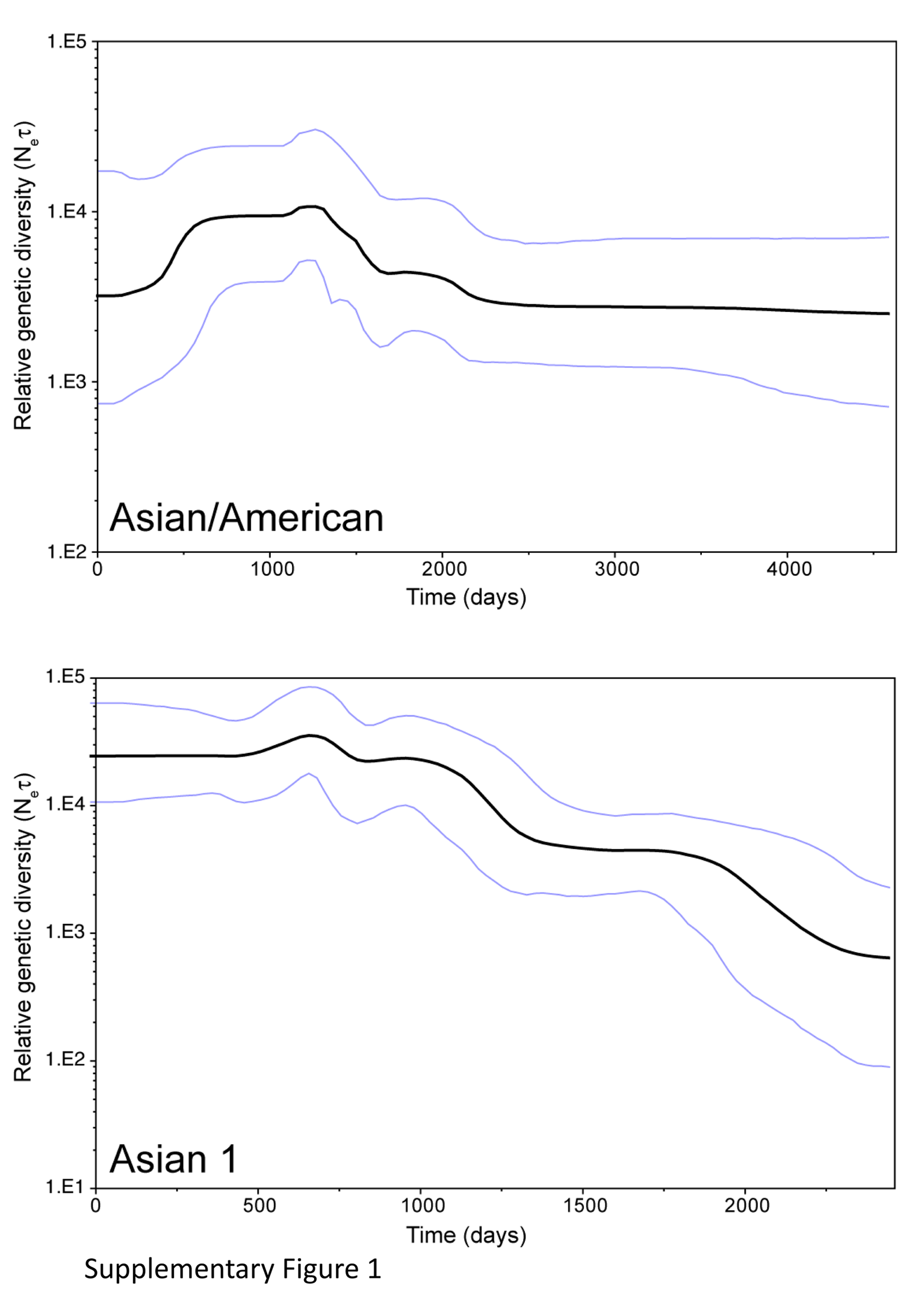

Supplement: Figure S1 — Bayesian skyline plots, showing changes in relatively genetic diversity through time (Neτ), for the Asian/American (n = 46) and Asian I (n = 139) genotypes in Viet Nam. Time (x-axis) is measured in days from the present (day 0), although it is important to note that different time-scales are used for each genotype. Note the decline of the Asian/American genotype correspondent to the rise of Asian I. (9.96 MB TIF) [file pntd.0000757.s001.tif]

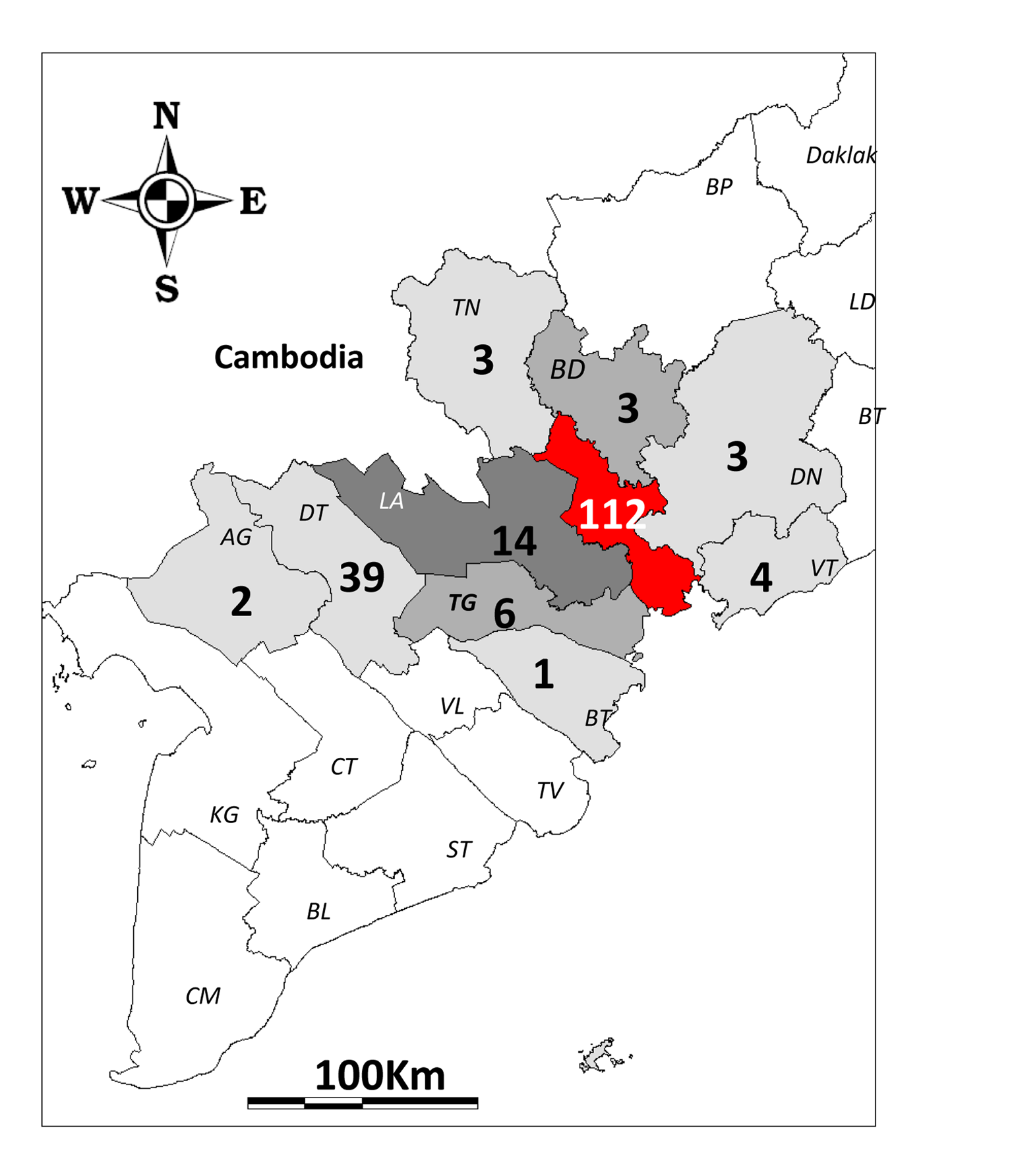

Supplement: Figure S2 — Map showing the provinces of southern Viet Nam. The numbers in each province represent the number of patients who reported living in that province at the time of admission to hospital and from whom a DENV-2 genome sequence was determined. (8.24 MB TIF) [file pntd.0000757.s002.tif]

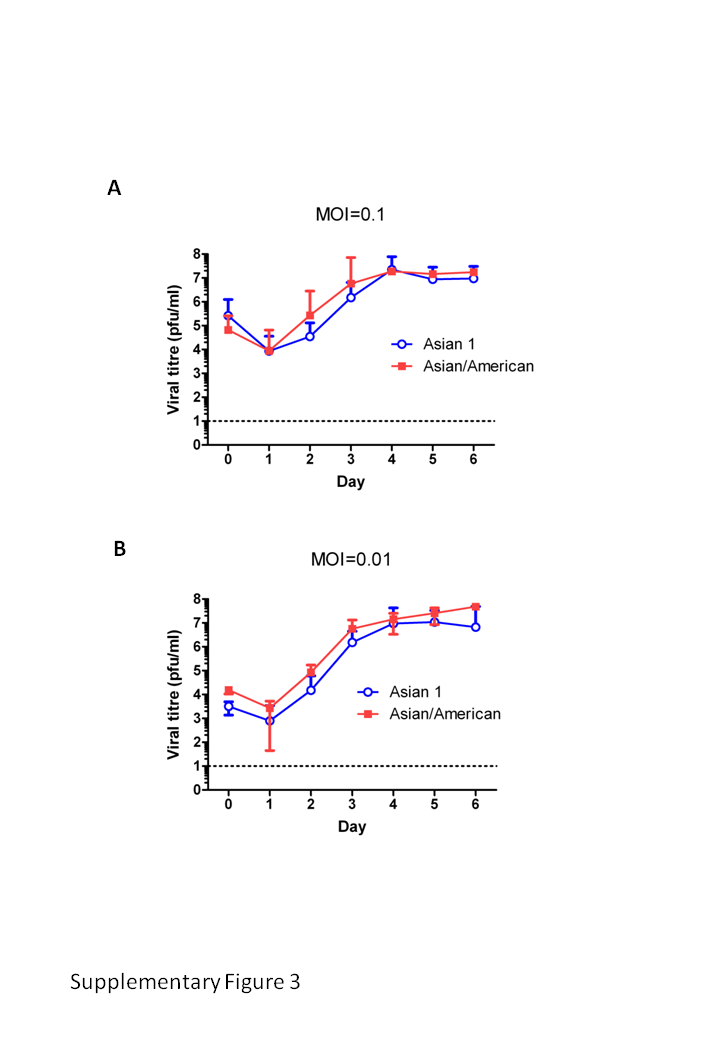

Supplement: Figure S3 — Growth kinetics of Asian/American and Asian 1 DENV-2 viruses in C6/36 cells. Three low-passage viruses representing Asian 1 (GenBank accession numbers: EU482542, EU482659, EU482654) and Asian/American genotype viruses (GenBank accession numbers: FM210221, FM210219, FM210221) were inoculated onto C6/36 cells in 2 ml tubes at multiplicities of infection of 0.1 and 0.01. Ten minutes after inoculation (time point = 0), and again every 24 hrs, the culture supernatant was sampled (100 microlitres) and the virus titre determined by plaque titration on BHK-21 cells. Shown are the mean and 95% confidence interval of the titre by day of collection. The dashed line represents the limit of detection. (0.11 MB TIF) [file pntd.0000757.s003.tif]
