# Supplementary material for: Emergence of the Asian 1 Genotype of Dengue Virus Serotype 2 in Viet Nam: In Vivo Fitness Advantage and Lineage Replacement in South-East Asia
Source: PLoS Negl Trop Dis. 2010 Jul 20;4(7):e757. doi: 10.1371/journal.pntd.0000757 (PMC2907417; doi:10.1371/journal.pntd.0000757)
Supplement: Table S1 — Genbank Accession numbers of DENV-2 genome sequences determined in this study. (0.22 MB DOC) [file pntd.0000757.s004.doc]

**Supplementary Table 1. Genbank Accession numbers of DENV-2 genome sequences determined in this study.**

| **Accession No** | **Serotype** | **Year of sampling** | **Country** |
| --- | --- | --- | --- |
| EU482640 | DENV2 | 2006 | Viet Nam |
| EU482641 | DENV2 | 2006 | Viet Nam |
| EU482642 | DENV2 | 2006 | Viet Nam |
| EU482643 | DENV2 | 2006 | Viet Nam |
| EU482644 | DENV2 | 2006 | Viet Nam |
| EU482645 | DENV2 | 2006 | Viet Nam |
| EU482646 | DENV2 | 2006 | Viet Nam |
| EU482647 | DENV2 | 2006 | Viet Nam |
| EU482648 | DENV2 | 2006 | Viet Nam |
| EU482649 | DENV2 | 2006 | Viet Nam |
| EU482650 | DENV2 | 2006 | Viet Nam |
| EU482651 | DENV2 | 2006 | Viet Nam |
| EU482652 | DENV2 | 2006 | Viet Nam |
| EU482653 | DENV2 | 2006 | Viet Nam |
| EU482654 | DENV2 | 2006 | Viet Nam |
| EU482655 | DENV2 | 2006 | Viet Nam |
| EU482656 | DENV2 | 2006 | Viet Nam |
| EU482657 | DENV2 | 2006 | Viet Nam |
| EU482658 | DENV2 | 2006 | Viet Nam |
| EU482659 | DENV2 | 2006 | Viet Nam |
| EU482660 | DENV2 | 2006 | Viet Nam |
| EU482661 | DENV2 | 2006 | Viet Nam |
| EU482662 | DENV2 | 2006 | Viet Nam |
| EU482663 | DENV2 | 2006 | Viet Nam |
| EU482664 | DENV2 | 2006 | Viet Nam |
| EU482665 | DENV2 | 2006 | Viet Nam |
| EU482666 | DENV2 | 2006 | Viet Nam |
| EU482667 | DENV2 | 2006 | Viet Nam |
| EU482668 | DENV2 | 2006 | Viet Nam |
| EU482669 | DENV2 | 2006 | Viet Nam |
| EU482670 | DENV2 | 2006 | Viet Nam |
| EU482671 | DENV2 | 2006 | Viet Nam |
| EU482672 | DENV2 | 2006 | Viet Nam |
| EU482673 | DENV2 | 2006 | Viet Nam |
| EU482674 | DENV2 | 2006 | Viet Nam |
| EU482675 | DENV2 | 2006 | Viet Nam |
| EU482676 | DENV2 | 2006 | Viet Nam |
| EU482677 | DENV2 | 2006 | Viet Nam |
| EU482678 | DENV2 | 2006 | Viet Nam |
| EU482679 | DENV2 | 2007 | Viet Nam |
| EU482463 | DENV2 | 2006 | Viet Nam |
| EU482541 | DENV2 | 2006 | Viet Nam |
| EU482542 | DENV2 | 2006 | Viet Nam |
| EU482543 | DENV2 | 2006 | Viet Nam |
| EU482464 | DENV2 | 2006 | Viet Nam |
| EU482445 | DENV2 | 2006 | Viet Nam |
| EU482446 | DENV2 | 2006 | Viet Nam |
| EU482466 | DENV2 | 2006 | Viet Nam |
| EU482467 | DENV2 | 2006 | Viet Nam |
| EU569721 | DENV2 | 2006 | Viet Nam |
| EU482468 | DENV2 | 2006 | Viet Nam |
| EU482447 | DENV2 | 2006 | Viet Nam |
| EU482448 | DENV2 | 2006 | Viet Nam |
| EU482449 | DENV2 | 2006 | Viet Nam |
| EU482450 | DENV2 | 2006 | Viet Nam |
| EU660413 | DENV2 | 2007 | Viet Nam |
| EU482469 | DENV2 | 2006 | Viet Nam |
| EU482470 | DENV2 | 2006 | Viet Nam |
| EU482465 | DENV2 | 2006 | Viet Nam |
| EU482451 | DENV2 | 2006 | Viet Nam |
| EU482471 | DENV2 | 2006 | Viet Nam |
| EU482697 | DENV2 | 2007 | Viet Nam |
| EU482698 | DENV2 | 2007 | Viet Nam |
| EU482699 | DENV2 | 2007 | Viet Nam |
| EU482700 | DENV2 | 2007 | Viet Nam |
| EU482475 | DENV2 | 2007 | Viet Nam |
| EU482701 | DENV2 | 2007 | Viet Nam |
| EU482702 | DENV2 | 2007 | Viet Nam |
| EU482703 | DENV2 | 2007 | Viet Nam |
| EU482704 | DENV2 | 2007 | Viet Nam |
| EU482705 | DENV2 | 2007 | Viet Nam |
| EU482474 | DENV2 | 2007 | Viet Nam |
| EU660414 | DENV2 | 2007 | Viet Nam |
| EU687248 | DENV2 | 2007 | Viet Nam |
| EU482473 | DENV2 | 2006 | Viet Nam |
| EU660415 | DENV2 | 2007 | Viet Nam |
| EU687249 | DENV2 | 2007 | Viet Nam |
| EU660416 | DENV2 | 2007 | Viet Nam |
| EU726776 | DENV2 | 2007 | Viet Nam |
| EU660417 | DENV2 | 2007 | Viet Nam |
| EU677137 | DENV2 | 2007 | Viet Nam |
| EU677138 | DENV2 | 2007 | Viet Nam |
| EU677148 | DENV2 | 2007 | Viet Nam |
| EU677149 | DENV2 | 2007 | Viet Nam |
| EU687250 | DENV2 | 2007 | Viet Nam |
| FJ410288 | DENV2 | 2007 | Viet Nam |
| FJ024452 | DENV2 | 2007 | Viet Nam |
| FJ205877 | DENV2 | 2007 | Viet Nam |
| FJ024454 | DENV2 | 2007 | Viet Nam |
| FJ390384 | DENV2 | 2007 | Viet Nam |
| FJ205878 | DENV2 | 2007 | Viet Nam |
| FJ205879 | DENV2 | 2007 | Viet Nam |
| FJ390385 | DENV2 | 2007 | Viet Nam |
| FJ024458 | DENV2 | 2007 | Viet Nam |
| FJ205880 | DENV2 | 2007 | Viet Nam |
| FJ390387 | DENV2 | 2007 | Viet Nam |
| FJ024461 | DENV2 | 2007 | Viet Nam |
| FJ373299 | DENV2 | 2007 | Viet Nam |
| FJ410193 | DENV2 | 2007 | Viet Nam |
| FJ410195 | DENV2 | 2007 | Viet Nam |
| FJ432724 | DENV2 | 2007 | Viet Nam |
| FJ432726 | DENV2 | 2007 | Viet Nam |
| FJ562098 | DENV2 | 2007 | Viet Nam |
| FJ859028 | DENV2 | 2007 | Viet Nam |
| FJ410200 | DENV2 | 2007 | Viet Nam |
| FJ410202 | DENV2 | 2007 | Viet Nam |
| FJ410208 | DENV2 | 2007 | Viet Nam |
| FJ461305 | DENV2 | 2007 | Viet Nam |
| FJ410215 | DENV2 | 2008 | Viet Nam |
| FJ461309 | DENV2 | 2008 | Viet Nam |
| FJ461311 | DENV2 | 2008 | Viet Nam |
| FJ410217 | DENV2 | 2007 | Viet Nam |
| FJ410219 | DENV2 | 2007 | Viet Nam |
| FJ461314 | DENV2 | 2007 | Viet Nam |
| FJ410221 | DENV2 | 2007 | Viet Nam |
| FJ547064 | DENV2 | 2007 | Viet Nam |
| FJ410223 | DENV2 | 2007 | Viet Nam |
| FJ410224 | DENV2 | 2007 | Viet Nam |
| FJ461321 | DENV2 | 2007 | Viet Nam |
| FJ410228 | DENV2 | 2007 | Viet Nam |
| FJ410233 | DENV2 | 2008 | Viet Nam |
| FJ410237 | DENV2 | 2008 | Viet Nam |
| FJ410241 | DENV2 | 2008 | Viet Nam |
| FJ410259 | DENV2 | 2008 | Viet Nam |
| FJ547067 | DENV2 | 2007 | Viet Nam |
| FJ873811 | DENV2 | 2006 | Viet Nam |
| EU482782 | DENV2 | 2003 | Viet Nam |
| EU482783 | DENV2 | 2003 | Viet Nam |
| EU482784 | DENV2 | 2003 | Viet Nam |
| EU482785 | DENV2 | 2003 | Viet Nam |
| EU482786 | DENV2 | 2003 | Viet Nam |
| EU482787 | DENV2 | 2003 | Viet Nam |
| EU482788 | DENV2 | 2003 | Viet Nam |
| EU482774 | DENV2 | 2004 | Viet Nam |
| EU482472 | DENV2 | 2004 | Viet Nam |
| EU482775 | DENV2 | 2004 | Viet Nam |
| EU482776 | DENV2 | 2005 | Viet Nam |
| EU482777 | DENV2 | 2005 | Viet Nam |
| EU482778 | DENV2 | 2003 | Viet Nam |
| EU482779 | DENV2 | 2003 | Viet Nam |
| EU482780 | DENV2 | 2003 | Viet Nam |
| EU482781 | DENV2 | 2003 | Viet Nam |
| FM210246 | DENV2 | 2005 | Viet Nam |
| FM210245 | DENV2 | 2005 | Viet Nam |
| FM210244 | DENV2 | 2004 | Viet Nam |
| FM210243 | DENV2 | 2004 | Viet Nam |
| FM210242 | DENV2 | 2004 | Viet Nam |
| FM210241 | DENV2 | 2004 | Viet Nam |
| FM210240 | DENV2 | 2004 | Viet Nam |
| FM210239 | DENV2 | 2004 | Viet Nam |
| FM210238 | DENV2 | 2001 | Viet Nam |
| FM210237 | DENV2 | 2003 | Viet Nam |
| FM210236 | DENV2 | 2004 | Viet Nam |
| FM210235 | DENV2 | 2005 | Viet Nam |
| FM210233 | DENV2 | 2004 | Viet Nam |
| FM210234 | DENV2 | 2004 | Viet Nam |
| FM210232 | DENV2 | 2004 | Viet Nam |
| FM210231 | DENV2 | 2004 | Viet Nam |
| FM210230 | DENV2 | 2003 | Viet Nam |
| FM210229 | DENV2 | 2003 | Viet Nam |
| FM210228 | DENV2 | 2003 | Viet Nam |
| FM210227 | DENV2 | 2002 | Viet Nam |
| FM210226 | DENV2 | 2002 | Viet Nam |
| FM210225 | DENV2 | 2001 | Viet Nam |
| FM210224 | DENV2 | 2001 | Viet Nam |
| FM210223 | DENV2 | 2003 | Viet Nam |
| FM210222 | DENV2 | 2004 | Viet Nam |
| FM210221 | DENV2 | 2003 | Viet Nam |
| FM210220 | DENV2 | 2003 | Viet Nam |
| FM210219 | DENV2 | 2003 | Viet Nam |
| FM210218 | DENV2 | 2002 | Viet Nam |
| FM210217 | DENV2 | 2004 | Viet Nam |
| FM210216 | DENV2 | 2004 | Viet Nam |
| FM210215 | DENV2 | 2004 | Viet Nam |
| FM210214 | DENV2 | 2004 | Viet Nam |
| FM210213 | DENV2 | 2005 | Viet Nam |
| FM210212 | DENV2 | 2003 | Viet Nam |
| FM210211 | DENV2 | 2003 | Viet Nam |
| FM210210 | DENV2 | 2003 | Viet Nam |
| FM210209 | DENV2 | 2003 | Viet Nam |
| FM210208 | DENV2 | 2003 | Viet Nam |
| FM210207 | DENV2 | 2005 | Viet Nam |
| FM210206 | DENV2 | 2005 | Viet Nam |
| FM210205 | DENV2 | 2005 | Viet Nam |
| FM210204 | DENV2 | 2003 | Viet Nam |
| FM210202 | DENV2 | 2004 | Viet Nam |
| FM210203 | DENV2 | 2003 | Viet Nam |
| GU131932 | DENV-2 | 2008 | Cambodia |
| GU131931 | DENV-2 | 2008 | Cambodia |
| GU131930 | DENV-2 | 2008 | Cambodia |
| GU131929 | DENV-2 | 2008 | Cambodia |
| GU131928 | DENV-2 | 2008 | Cambodia |
| GU131927 | DENV-2 | 2007 | Cambodia |
| GU131924 | DENV-2 | 2008 | Cambodia |
| GU131902 | DENV-2 | 2008 | Cambodia |
| GU131901 | DENV-2 | 2008 | Cambodia |
| GU131900 | DENV-2 | 2008 | Cambodia |
| GU131899 | DENV-2 | 2008 | Cambodia |
| GU131898 | DENV-2 | 2008 | Cambodia |
| GU131897 | DENV-2 | 2007 | Cambodia |
| GU131896 | DENV-2 | 2007 | Cambodia |
| GQ868638 | DENV-2 | 2008 | Cambodia |
| GQ868631 | DENV-2 | 2008 | Cambodia |
| GQ868625 | DENV-2 | 2008 | Cambodia |
| GQ868624 | DENV-2 | 2007 | Cambodia |
| GQ868623 | DENV-2 | 2005 | Cambodia |
| GQ868622 | DENV-2 | 2003 | Cambodia |
| GQ868621 | DENV-2 | 2003 | Cambodia |
| GQ868620 | DENV-2 | 2003 | Cambodia |
| FJ639718 | DENV-2 | 2008 | Cambodia |
| FJ639717 | DENV-2 | 2007 | Cambodia |
| FJ639711 | DENV-2 | 2005 | Cambodia |
| FJ639710 | DENV-2 | 2005 | Cambodia |
| FJ639709 | DENV-2 | 2005 | Cambodia |
| FJ639708 | DENV-2 | 2005 | Cambodia |
| FJ639707 | DENV-2 | 2004 | Cambodia |
| FJ639706 | DENV-2 | 2004 | Cambodia |
| FJ639705 | DENV-2 | 2003 | Cambodia |
| FJ639704 | DENV-2 | 2003 | Cambodia |
| FJ639703 | DENV-2 | 2003 | Cambodia |
| FJ639701 | DENV-2 | 2002 | Cambodia |
| FJ639700 | DENV-2 | 2002 | Cambodia |
| FJ639699 | DENV-2 | 2002 | Cambodia |
| FJ639698 | DENV-2 | 2002 | Cambodia |
| FJ639697 | DENV-2 | 2001 | Cambodia |
| FJ639702 | DENV-2 | 2003 | Cambodia |
